# Light at night disrupts diel patterns of cytokine gene expression and endocrine profiles in zebra finch (*Taeniopygia guttata*)

**DOI:** 10.1038/s41598-019-51791-9

**Published:** 2019-11-01

**Authors:** Ila Mishra, Reinhard M. Knerr, Alexander A. Stewart, Wesley I. Payette, Melanie M. Richter, Noah T. Ashley

**Affiliations:** Department of Biology, Western Kentucky University, Bowling Green, KY USA

**Keywords:** Ecophysiology, Interleukins

## Abstract

Increased exposure to light pollution perturbs physiological processes through misalignment of daily rhythms at the cellular and tissue levels. Effects of artificial light-at-night (ALAN) on diel properties of immunity are currently unknown. We therefore tested the effects of ALAN on diel patterns of cytokine gene expression, as well as key hormones involved with the regulation of immunity, in zebra finches (*Taeniopygia guttata*). Circulating melatonin and corticosterone, and mRNA expression levels of pro- (*IL-1β*, *IL-6*) and anti-inflammatory (*IL-10*) cytokines were measured at six time points across 24-h day in brain (nidopallium, hippocampus, and hypothalamus) and peripheral tissues (liver, spleen, and fat) of zebra finches exposed to 12 h light:12 h darkness (LD), dim light-at-night (DLAN) or constant bright light (LLbright). Melatonin and corticosterone concentrations were significantly rhythmic under LD, but not under LLbright and DLAN. Genes coding for cytokines showed tissue-specific diurnal rhythms under LD and were lost with exposure to LLbright, except *IL-6* in hypothalamus and liver. In comparison to LLbright, effects of DLAN were less adverse with persistence of some diurnal rhythms, albeit with significant waveform alterations. These results underscore the circadian regulation of biosynthesis of immune effectors and imply the susceptibility of daily immune and endocrine patterns to ALAN.

## Introduction

Nearly all vertebrate species on earth synchronize their daily and annual activities based on information from the sun^1^. This daily alignment of behavior and physiology to photic cues is regulated by the response of circadian machinery to light:dark (LD) cycles. In mammals, the master circadian clock resides in the suprachiasmatic nucleus (SCN) of the hypothalamus^2^. Among birds, a more complex circadian system of three pacemakers viz. the avian equivalent of the SCN in hypothalamus, pineal gland, and the retina regulate daily timekeeping^3^.

The worldwide increase in artificial lighting in the past century had led to alterations in circadian- and seasonally-controlled behavior and physiology of many vertebrates, including birds^4–6^. For example, in songbirds, artificial light at night (ALAN) and constant exposure to light (LL) have been shown to impact singing, sleep, cognitive performance, personality, and reproduction^7–11^. More specifically, great tits, *Parus major*, when exposed to dim light at night (DLAN), sleep less and spend less time at nest boxes^7^, while Indian house crows, *Corvus splendens,* exposed to LL spent significantly more time to correctly perform memory tasks^10^. Timing of circannual gonadal recrudescence in spotted munia, *Lonchura punctulate*, is also altered in LL and under aberrant LD cycles of 3.5 h light: 3.5 h darkness^11^. Alterations in avian circadian clocks are also reported in response to ALAN^12^ and LL^13^.

An emerging finding in the mammalian literature is that the immune system is also influenced by circadian function and thus susceptible to ALAN^14^. Similarly, several avian studies have demonstrated susceptibility of immune responses to circadian dysfunction and sleep loss^15,16^. For example, while ALAN increased oxidative stress in great tit nestlings^16^, exposure to extended hours of light and LL suppressed several measures of adaptive immunity (humoral and cell-mediated immunity) in broiler chickens, *Gallus gallus*^17^ and quail, *Coturnix japonica*^18^. Other studies have shown effects of circadian disruption upon innate immune function, namely inflammatory markers, as measured by pro-inflammatory cytokine gene expression in brain and peripheral tissues. More specifically, sleep loss increased pro-inflammatory cytokine gene expression in brain, adipose tissue, and spleen of zebra finches, *Taeneopygia guttata*^19^. Cytokine gene expression in the avian spleen, a commonly used indicator of immune responses^20^, was significantly altered under conditions of LL and melatonin supplementation^21^. Furthermore, the avian spleen displayed rhythmic oscillations of cytokine mRNA abundance over a 24 h cycle as well as a daily rhythm in response to lipopolysaccharide (LPS)-induced immune challenge when applied at midday vs. midnight^21^. An upregulation of proinflammatory cytokines in adipose tissue from sleep disturbances or LD cycle perturbations may be attributed to the infiltration of classical proinflammatory M1 macrophages into adipose tissue^22^. In brain, light-induced changes in expression levels of cytokines can affect several aspects of normal central nervous system functioning, including sleep, synaptic pruning, and regulation of a variety of neuroendocrine functions in addition to neuroinflammation^23^. In general, brain pro-inflammatory cytokines approach maximum levels at night during periods of increased slow-wave sleep^24^. Further, Kupffer cells in liver are also able to synthesize a variety of cytokines and an upregulation of hepatic pro-inflammatory response can have systemic effects on other organs or can alter hepatic metabolic function through paracrine action^25^.

The effects of circadian disruption upon immunity could be mediated through several endocrine pathways that include melatonin, thyroid hormones, testosterone, glucocorticoids, and their interactions^26^. Melatonin is an indolamine primarily secreted by the pineal gland at night and suppressed by light during the day^27^. Melatonin is typically immunoenhancing in birds, as indicated by studies in Japanese quail^28^, jungle bush quail (*Perdicula asiatica*^29,30^) and chicken^31^. Partially-abolished diurnal rhythms in *IL-6* and *IL-18* mRNA expression under LL were restored by melatonin supplementation in chickens^31^. On the other hand, stimulatory effects of melatonin on cellular and humoral immune response in quail are dependent on opioids^18^, while modulation of seasonal immunity in Indian tropical birds, *Perdicula asiatica*, involves an interaction between melatonin and sex steroids with melatonin enhancing and sex steroids suppressing immunity^32^. In addition, plasma corticosterone (cort) concentration follows a daily rhythm with the peak close to sunrise in diurnal species, such as Gambel’s white crowned sparrows (*Zonotrichia leucophrys gambelii*)^33^. In contrast, nocturnal bats exhibit peak plasma cort concentrations around sunset^34^. Free-living great tits breeding in LAN had higher cort levels than those breeding under control sites^35^. Further, effects of LAN on glucocorticoid responses can be light temperature-dependent. Zebra finches exposed to 5000 K LAN had increased corticosterone levels compared to finches exposed to 3000 K^36^. Exposure to LL or LAN may alter the immune response through activation of the hypothalamic-pituitary-adrenal (HPA) axis^26^. Acute elevation in cort levels in response to short-term stress can enhance immune function, while chronic activation of the HPA axis may have opposite effects^37^.

While many mammalian studies report diurnal rhythms in immune responses, including expression profile of cytokines in brain and peripheral tissues^37,38^, less is known in birds. Recent studies involving LAN protocols offer clues to circadian regulation of immune response in birds; however, the effects of circadian disruption on daily oscillations in immune function are largely unknown. The aims of this study are to (a) elucidate the diurnal variation of peripheral and neural expression of cytokine genes over the 24-hour cycle, and (b) to test whether 10-days of exposure to constant light or DLAN affects rhythmicity of cytokine gene expression, and/or the profiles of key endocrine regulators of immunity. Given the varied functions of cytokines across tissues^22,23,25^, we hypothesize that diurnal variation in cytokines will follow a tissue-specific expression pattern and that these diurnal waveforms will be susceptible to light-dark cycle perturbations. We also hypothesize that cytokine gene expression will peak mostly during the day in various tissues to coincide with increased activity and higher disease risk, although some cytokines in brain could peak at night to modulate sleep. We also predict that endocrine profiles will be affected by different light treatments and could potentially modulate rhythmicity of cytokine gene expression. For example, if constant light and DLAN treatments suppress melatonin rhythms and/or alter the amplitude of cort rhythms on a chronic level, then we would predict disruption of constitutive rhythms of cytokine gene expression. Given previous studies, we also predicted that DLAN would disrupt some (but not all) of the diel rhythms in immune function as opposed to complete abolishment of rhythms from constant light.

## Results

### 24-hour variation in hormone levels

**Figure 1:**
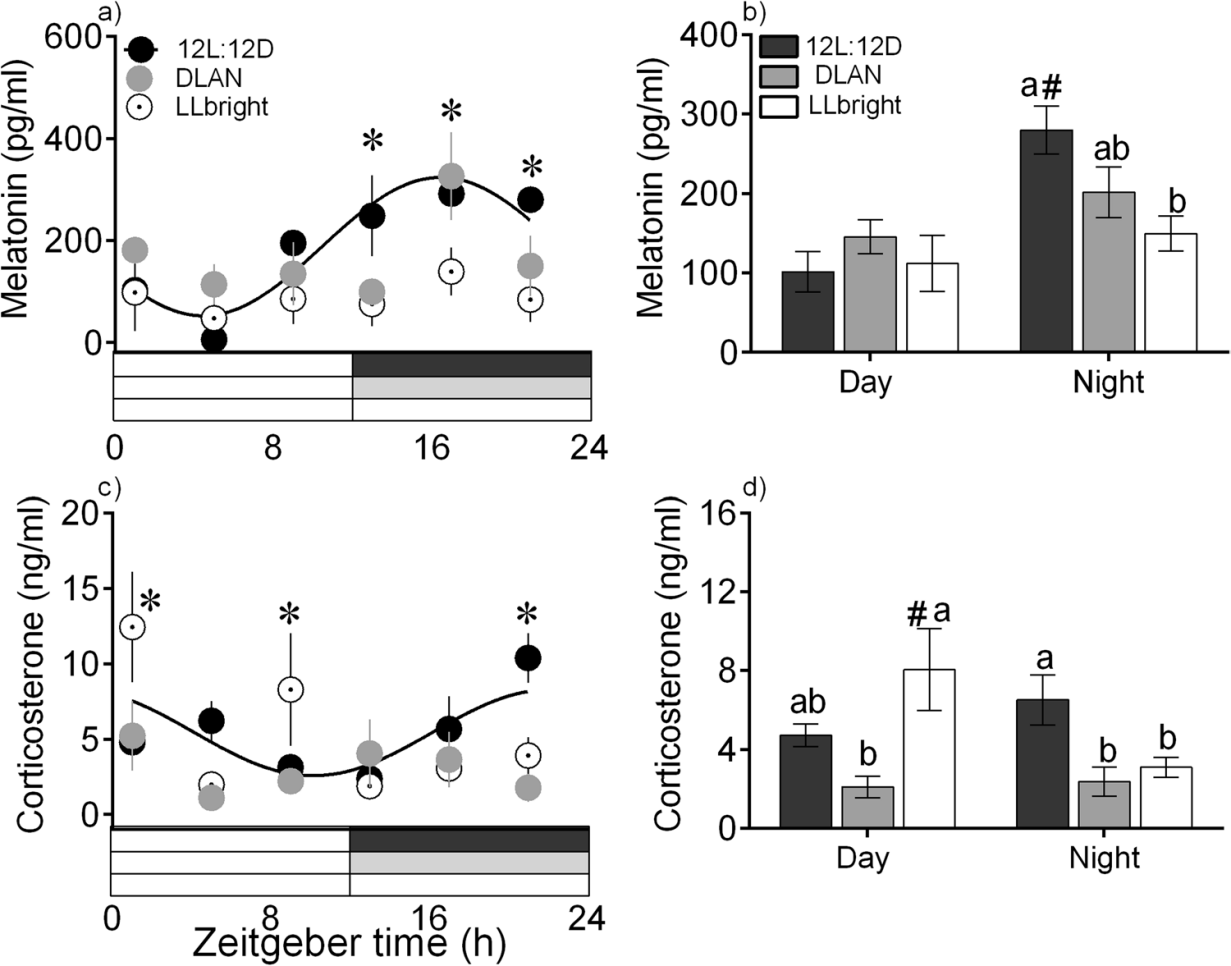
Mean (±SE) levels of melatonin (**a**,**b**) and corticosterone (**c**,**d**) measured at ZT 1, 5, 9, 13, 17 and (Zeitgeber time 0 (ZT 0) = lights on, n = 5/time-point) in plasma and serum, respectively of zebra finches exposed to 12 L:12D (solid black circles), DLAN (grey solid circles) and LLbright (white solid circles). Solid black curve indicates a significant 24-h rhythm in hormone levels in 12 L:12D, as determined by cosinor analysis. a, c: (*) Asterisk over time points in 24-h profile indicate significant differences between LD cycles. b, d: (#) present significant difference between the day and night levels. Different and same alphabets present significant and no difference between LD conditions, respectively, as determined by Bonferroni post-hoc tests following 2-way ANOVA. *p* < 0.05 was considered statistically significant.

Serum melatonin and corticosterone levels significantly varied across the 24-h day, as determined by two-way ANOVA (n = 5/time point, effect of time-of-day; Table 1, Suppl. Table 1), and both hormones exhibited significant daily rhythm under 12 L:12D (F-test, Fig. 1a,c), with peak levels occurring early (hour 19) and late (hour 22) during the night, respectively (Cosinor analysis, Tables 2, 3, Suppl. Table 2). There was a significant effect of LD cycle (2-way ANOVA Table 1, Suppl. Table 1) and an absence of a significant diurnal rhythm under DLAN and LLbright (Cosinor analysis, Tables 2, 3). The night (ZT 13, 17 and 21) levels of melatonin were significantly higher in 12 L:12D than the other two groups (except DLAN at ZT 17; Bonferroni post-hoc test, Fig. 1a). In addition, melatonin levels towards the middle of night (ZT 17) in DLAN were significantly higher than those in LLbright (Bonferroni post-hoc test, *p* < 0.05, Fig. 1a). Cort day-levels (ZT 1 and 9) were significantly higher under LLbright than DLAN and 12 L:12D (only at ZT 1), and late night-levels (ZT 21) were significantly higher in 12 L:12D than other groups (Bonferroni post-hoc test, *p* < 0.05, Fig. 1c).

**Table 1:** Statistical values of two-way analysis of variance (2-way ANOVA) testing the effects of light condition (factor 1), time-of-day (factor 2) and their interaction (factor 1 × 2) on the plasma hormone and tissue-specific gene expression levels.

Tissue	Hormone/gene	2-way ANOVA		
		LD condition	Time-of-day	Interaction
Blood	Melatonin	*F*_2,*7*2_ = 6.22, *p* = 0.003	*F*_*5*,*7*2_ = 3.49, *p* = 0.007	NS
	Cort	*F*_2,*7*2_ = 3.81, *p* = 0.027	*F*_*5*,*7*2_ = 2.99, *p* = 0.016	*F*_*10*,*7*2_ = 3.28, *p* = 0.002
Nidopallium	*IL-1β*	*F*_2,*7*2_ = 15.43, *p* < 0.0001	NS	*F*_*10*,*7*2_ = 3.51, *p* = 0.008
	*IL-6*	*F*_2,*7*2_ = 30.23, *p* < 0.0001	*F*_*5*,*7*2_ = 5.25, *p* < 0.0001	NS
	*IL-10*	*F*_2,*7*2_ = 34.82, *p* < 0.0001	*F*_*5*,*7*2_ = 5.67, *p* = 0.0002	*F*_*10*,*7*2_ = 5.12, *p* < 0.0001
Hippocampus	*IL-1β*	*F*_2,*7*2_ = 64.51, *p* < 0.0001	*F*_*5*,*7*2_ = 2.44, *p* = 0.042	*F*_*10*,*7*2_ = 2.39, *p* = 0.017
	*IL-6*	*F*_2,*7*2_ = 24.42, *p* < 0.0001	*F*_*5*,*7*2_ = 3.09, *p* = 0.014	*F*_*10*,*7*2_ = 2.79, *p* = 0.058
	*IL-10*	*F*_*2*,*66*_ = 45.25, *p* < 0.0001	*F*_*5*,*66*_ = 6.48, *p* < 0.0001	*F*_*10*,*66*_ = 5.58, *p* < 0.0001
Hypothalamus	*IL-1β*	*F*_*2*,*72*_ = 17.79, *p* < 0.0001	*F*_*5*,*72*_ = 3.11, *p* = 0.014	*F*_*10*,*72*_ = 2.46, *p* = 0.014
	*IL-6*	*F*_*2*,*72*_ = 35.43, *p* < 0.0001	*F*_*5*,*72*_ = 3.84, *p* = 0.004	*F*_*10*,*72*_ = 4.29, *p* < 0.0001
	*IL-10*	*F*_*2*,*72*_ = 57.30, *p* < 0.0001	*F*_*5*,*72*_ = 21.26, *p* < 0.0001	*F*_*10*,*72*_ = 23.3, *p* < 0.0001
Liver	*IL-1β*	*F*_*2*,*70*_ = 38.06, *p* < 0.0001	NS	*F*_*10*,*70*_ = 3.05, *p* = 0.003
	*IL-6*	*F*_*2*,*72*_ = 6.82, *p* = 0.002	*F*_*5*,*72*_ = 7.82, *p* < 0.0001	*F*_*10*,*72*_ = 8.55, *p* < 0.0001
	*IL-10*	*F*_*2*,*72*_ = 8.11, *p* = 0.0007	*F*_*5*,*72*_ = 6.49, *p* < 0.0001	*F*_*10*,*72*_ = 7.42, *p* < 0.0001
Spleen	*IL-1β*	*F*_*2*,*72*_ = 3.44, *p* = 0.037	*F*_*5*,*72*_ = 3.66, *p* = 0.005	*F*_*10*,*72*_ = 3.11, *p* = 0.002
	*IL-6*	*F*_*2*,*72*_ = 11.86, *p* < 0.0001	*F*_*5*,*72*_ = 9.60, *p* < 0.0001	*F*_*10*,*72*_ = 4.89, *p* < 0.0001
	*IL-10*	*F*_*2*,*72*_ = 13.45, *p* < 0.0001	*F*_*5*,*72*_ = 6.09, *p* < 0.0001	*F*_*10*,*72*_ = 7.75, *p* < 0.0001
Fat	*IL-1β*	*F*_*2*,*72*_ = 9.63, *p* = 0.002	*F*_*5*,*72*_ = 2.44, *p* = 0.042	*F*_*10*,*72*_ = 2.48, *p* = 0.0001
	*IL-6*	*F*_*2*,*72*_ = 42.18, *p* < 0.0001	*F*_*5*,*72*_ = 3.53, *p* = 0.006	*F*_*10*,*72*_ = 2.12, *p* = 0.034
	*IL-10*	*F*_*2*,*72*_ = 33.06, *p* < 0.0001	NS	*F*_*10*,*72*_ = 7.19, *p* < 0.0001

NS (not significant) indicates an absence of significant effect. *p* < 0.05 was considered statistically significant.

**Table 2:** A significant diurnal waveform tested using the number of samples, r^2^ values, and number of predictors -mesor, amplitude, and acrophase.

Tissue	Hormone/gene	12 L:12D	DLAN	LLbright
Blood	Melatonin	*r*^*2*^ = 0.49, *F* = 8.15, *p* = 0.0005	NR	NR
	Cort	*r*^*2*^ = 0.30, *F* = 3.71, *p* = 0.023	NR	NR
Nidopallium	*IL-1β*	NR	NR	NR
	*IL-6*	*r*^*2*^ = 0.31, *F* = 3.93, *p* = 0.019	*r*^*2*^ = 0.32, *F* = 3.82, *p* = 0.022	NR
	*IL-10*	*r*^*2*^ = 0.44, *F* = 6.71, *p* = 0.001	NR	NR
Hippocampus	*IL-1β*	*r*^*2*^ = 0.39, *F* = 5.41, *p* = 0.005	NR	NR
	*IL-6*	*r*^*2*^ = 0.32, *F* = 4.06, *p* = 0.017	*r*^*2*^ = 0.32, *F* = 4.04, *p* = 0.017	NR
	*IL-10*	NR	*r*^*2*^ = 0.37, *F* = 5.27, *p* = 0.006	NR
Hypothalamus	*IL-1β*	NR	NR	NR
	*IL-6*	*r*^*2*^ = 0.31, *F* = 3.97, *p* = 0.018	NR	*r*^*2*^ = 0.45, *F* = 7.09, *p* = 0.001
	*IL-10*	*r*^*2*^ = 0.31, *F* = 3.91, *p* = 0.019	NR	NR
Liver	*IL-1β*	*r*^*2*^ = 0.37, *F* = 5.09, *p* = 0.006	*r*^*2*^ = 0.32, *F* = 4.15, *p* = 0.016	NR
	*IL-6*	*r*^*2*^ = 0.30, *F* = 3.79, *p* = 0.022	*r*^*2*^ = 0.34, *F* = 4.37, *p* = 0.013	*r*^*2*^ = 0.33, *F* = 4.35, *p* = 0.013
	*IL-10*	*r*^*2*^ = 0.36, *F* = 4.85, *p* = 0.008	*r*^*2*^ = 0.30, *F* = 3.71, *p* = 0.024	NR
Spleen	*IL-1β*	*r*^*2*^ = 0.39, *F* = 5.05, *p* = 0.007	NR	NR
	*IL-6*	*r*^*2*^ = 0.57, *F* = 11.6, *p* < 0.0001	NR	NR
	*IL-10*	NR	NR	NR
Fat	*IL-1β*	*r*^*2*^ = 0.34, *F* = 4.41, *p* = 0.012	*r*^*2*^ = 0.45, *F* = 7.06, *p* = 0.001	NR
	*IL-6*	*r*^*2*^ = 0.37, *F* = 5.09, *p* = 0.007	*r*^*2*^ = 0.34, *F* = 4.46, *p* = 0.012	NR
	*IL-10*	NR	NR	NR

NR (not rhythmic) indicates an absence of significant daily rhythm. *p* < 0.05 was considered statistically significant.

**Table 3:** Mean (±SE) rhythm waveform characteristics in brain (nidopallium, hippocampus, hypothalamus) and peripheral (liver, spleen and fat) tissues of zebra finches under different light-dark conditions, as determined by cosinor analysis.

Tissue	gene	12 L:12D			DLAN			LLbright		
		Mesor	Amplitude	Acrophase	Mesor	Amplitude	Acrophase	Mesor	Amplitude	Acrophase
Blood	*Melatonin*	190.9 ± 18.49	139.2 ± 26.15	18.89 ± 0.76	NR	NR	NR	NR	NR	NR
	*Cort*	5.44 ± 0.39	2.86 ± 0.84	22.16 ± 1.12	NR	NR	NR	NR	NR	NR
Nidopallium	*IL-1β*	NR	NR	NR	NR	NR	NR	NR	NR	NR
	*IL-6*	0.83 ± 0.07	0.35 ± 0.10	19.2 ± 1.09	0.72 ± 0.08	0.39 ± 0.11	6.24 ± 1.11	NR	NR	NR
	*IL-10*	0.35 ± 0.05	0.28 ± 0.07	20.62 ± 0.92	NR	NR	NR	NR	NR	NR
Hippocampus	*IL-1β*	1.03 ± 0.04	0.25 ± 0.06	10.23 ± 0.93	NR	NR	NR	NR	NR	NR
	*IL-6*	0.18 ± 0.03	0.16 ± 0.05	1.01 ± 1.07	0.69 ± 0.07	0.39 ± 0.11	17.13 ± 1.06	NR	NR	NR
	*IL-10*	NR	NR	NR	0.32 ± 0.04	0.24 ± 0.05	1.81 ± 0.95	NR	NR	NR
Hypothalamus	*IL-1β*	NR	NR	NR	NR	NR	NR	NR	NR	NR
	*IL-6*	0.16 ± 0.05	0.24 ± 0.07	23.43 ± 1.01	NR	NR	NR	0.82 ± 0.08	0.54 ± 0.12	18.9 ± 0.81
	*IL-10*	3.23 ± 0.73	3.59 ± 1.03	20.56 ± 1.09	NR	NR	NR	NR	NR	NR
Liver	*IL-1β*	0.53 ± 0.04	0.21 ± 0.05	6.35 ± 0.96	0.18 ± 0.02	0.08 ± 0.02	14.26 ± 1.06	NR	NR	NR
	*IL-6*	0.57 ± 0.07	0.34 ± 0.09	12.8 ± 1.11	0.54 ± 0.05	0.26 ± 0.07	14.58 ± 1.03	0.97 ± 0.18	0.96 ± 0.26	20.07 ± 1.03
	*IL-10*	1.18 ± 0.2	1.11 ± 0.28	18.9 ± 0.98	0.95 ± 0.19	0.91 ± 0.27	16.05 ± 1.15	NR	NR	NR
Spleen	*IL-1β*	0.27 ± 0.04	0.24 ± 0.59	4.11 ± 0.96	NR	NR	NR	NR	NR	NR
	*IL-6*	0.29 ± 0.05	0.42 ± 0.07	3.13 ± 0.63	NR	NR	NR	NR	NR	NR
	*IL-10*	NR	NR	NR	NR	NR	NR	NR	NR	NR
Fat	*IL-1β*	0.39 ± 0.03	0.17 ± 0.05	22.08 ± 1.01	0.76 ± 0.09	0.62 ± 0.13	11.47 ± 0.81	NR	NR	NR
	*IL-6*	0.18 ± 0.15	0.08 ± 0.02	11.53 ± 0.96	0.62 ± 0.04	0.23 ± 0.06	10.77 ± 1.02	NR	NR	NR
	*IL-10*	NR	NR	NR	NR	NR	NR	NR	NR	NR

A significant cosinor waveform tested using the number of samples, r^2^ values, and number of predictors -mesor, amplitude, and acrophase. NR (not rhythmic) indicates an absence of significant daily rhythm. *p* < 0.05 was considered statistically significant.

**Table 4:** Details of RT-PCR probes used for quantification of gene expression.

Name	Dye	ID	Context Sequence
*PPIA*	VIC	AIRSBD1	ACGGTTCNCAGTTCTTCATCTGCACTGCCAAGACTGAGTGGCTGGATGGC
*IL-1β*	FAM	AIVI5WP	TTCTTGGATGATATTTTCGAGCCCGTCTCCTTCCGGTGCATCAGAGGCAG
*IL-6*	FAM	AIT97QH	GTACCATAAGACAGATGGTGATCAATCCCGAAGAAGTGATCATTCCAGAT
*IL-10*	FAM	AIS09J9	CTGCTGGAGGAAATCAAGGGCAGGCTCGGCTGCCAGTCGGTGTCGGAGAT

Furthermore, a comparison of all day-time samples (ZT 1, 5, 9) with all night-time samples (ZT 13, 17, 21) showed that day/night variation in melatonin and cort levels was dependent on the LD cycle (interaction: melatonin- *F*_2,77_ = 3.78, *p* = 0.027; cort- *F*_2,81_ = 5.30, *p* = 0.0069), with significantly higher melatonin levels in the night of 12 L:12D than the day of 12 L:12D and the night of LLbright (Bonferroni post-hoc test, *p* < 0.05, Fig. 1b). Day levels of cort in LLbright were significantly higher than night levels in LLbright and day levels in DLAN, and night levels in 12 L:12D were significantly higher than night levels of other groups (Bonferroni post-hoc test, *p* < 0.05, Fig. 1d).

### 24-hour variation in interleukin mRNA expression in brain

**Figure 2:**
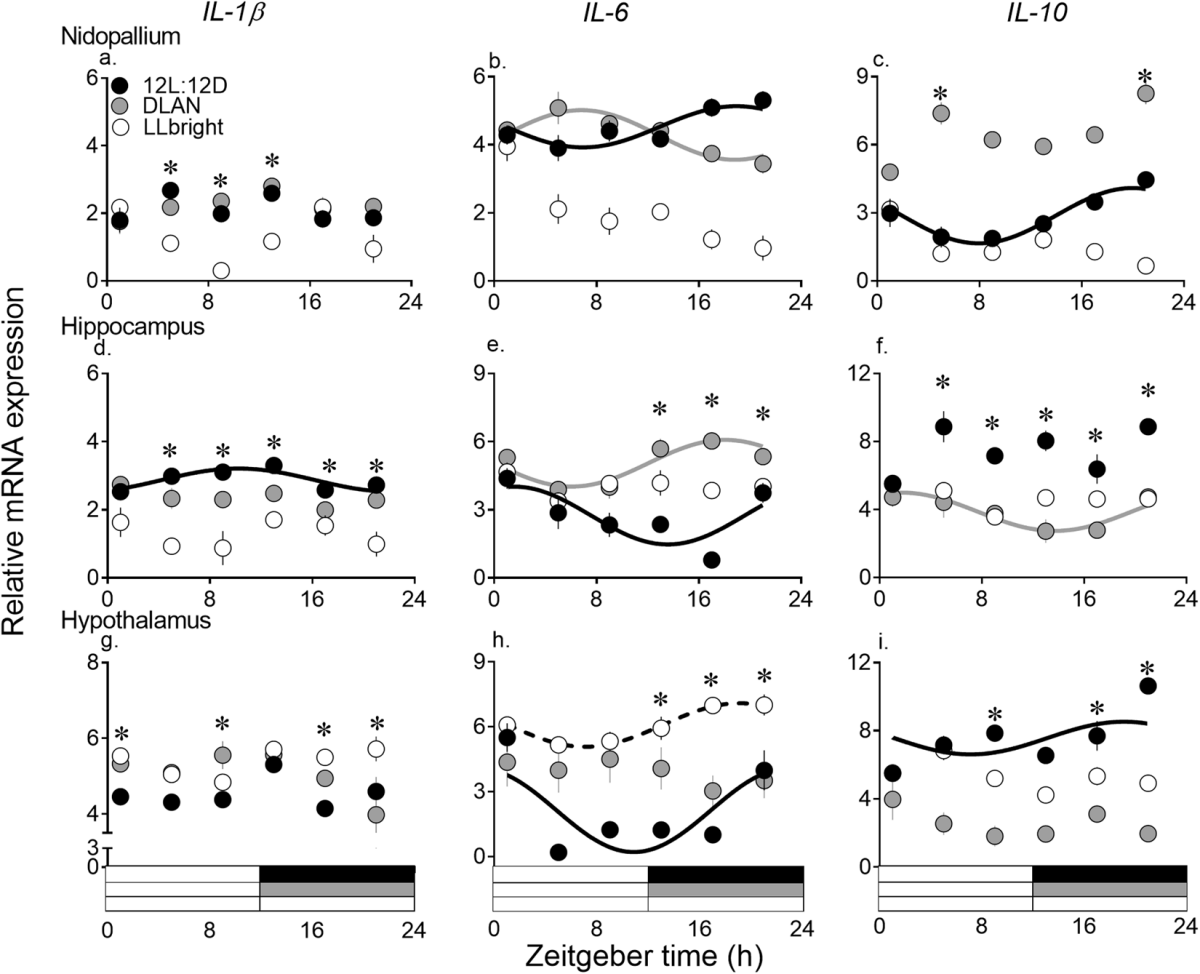
Mean (±SE) mRNA expression levels of genes coding for interleukins (*IL-1β*, *IL-6* and *IL-10*) measured at ZT1, 5, 9, 13, 17 and 21 (Zeitgeber time 0 (ZT 0) = lights on; n = 5/time-point) in nidopallium (**a**–**c**), hippocampus (**d**–**f**) and hypothalamus (**g**–**i**) of zebra finches exposed to 12 L:12D (solid black circles), DLAN (grey solid circles) and LLbright (white solid circles). Solid black, grey and dotted black curves indicate a significant 24-h rhythm under 12 L:12D, DLAN and LLbright, respectively, as determined by cosinor analysis. (*) Asterisk over time points indicate significant difference in gene expression under DLAN and/or LL from 12 L:12D, as determined by Bonferroni post-hoc test following a significant LD cycle x time-of-day interaction effect determined by 2-way ANOVA. *p* < 0.05 was considered statistically significant.

Cosinor analysis revealed a significant daily oscillation of *IL-6* in all three brain regions, of *IL-1β* in hippocampus, and of *IL-10* in nidopallium and hypothalamus under 12 L:12D (F test, *p* < 0.05, Fig. 2, Table 2). Under 12 L:12D, hippocampal *IL-6* and *IL-1β* peaked early (ZT 1) and late during the day (ZT 10), respectively, while *IL-6* and *IL-10* in nidopallium and hypothalamus peaked later during the night (ZT 19 to 23; Cosinor analysis, Fig. 2, Tables 2, 3). Light pollution significantly affected the neuroinflammatory diel patterns. All genes were arrhythmic under LLbright, except hypothalamic *IL-6* which peaked at ZT 19 (Cosinor analysis, Fig. 2h, Tables 2, 3). With DLAN exposure, there was a loss of daily rhythm in expression levels of all genes, except *IL-6* in nidopallium and hippocampus, and *IL-10* in hippocampus (Cosinor analysis, Fig. 2, Tables 2, 3). Further, DLAN significantly altered the waveform, with a shift of acrophase of *IL-6* from late night to middle of the day (ZT 6) in nidopallium, early in the day to early in the night (ZT 17) in hippocampus, and an acrophase of *IL-10* at ZT 2 in hippocampus (Cosinor analysis, Fig. 2, Table 3).

Overall, effects of LLbright and DLAN on interleukin mRNA expression were time-of-day dependent, as determined by a significant effect of LD x time-of-day interaction on *IL-1β*, *IL-10* and IL-6 (except in nidopallium) (2-way ANOVA; Table 1; Suppl Table 1). In nidopallium, LLbright and DLAN exposure significantly attenuated the pro-inflammatory (*IL-1β*) and anti-inflammatory response (*IL-10*), respectively. In comparison to 12 L:12D, LLbright significantly decreased *IL-1β* expression at ZT 5, 9 and 13 and DLAN significantly increased *IL-10* expression at ZT 5 and 21 (Bonferroni post-hoc test, *p* < 0.05, Fig. 2a–c). As opposed to nidopallium, light pollution significantly increased pro-inflammatory and decreased anti-inflammatory response in hypothalamus. LLbright exposure significantly increased *IL-6* expression during night (ZT 13, 17 and 21) and *IL-1β* expression early in the day (ZT 1) and later in the night (ZT 17 and 21; Bonferroni post-hoc test, *p* < 0.05, Fig. 2g,h). DLAN exposure increased *IL-1β* expression later in the day (ZT 9; Bonferroni post-hoc test, *p* < 0.05, Fig. 2g). On the other hand, hypothalamic *IL-10* levels were significantly higher in 12 L:12D at ZT 9, 17 and 21 relative to DLAN and LLbright (not at ZT 9; Bonferroni post-hoc test, *p* < 0.05, Fig. 2i). In hippocampus, LLbright exposure significantly decreased *IL-1β* and *IL-10* expression levels all throughout the day (except ZT 1; Bonferroni post-hoc test, *p* < 0.01, Fig. 2d,f), DLAN exposure significantly decreased *IL-1β* towards light-dark transition times (ZT 9, 13 and 17) and *IL-10* during the night (ZT 13, 17 and 21) and at middle of the day (ZT 5; Bonferroni post-hoc test, *p* < 0.01, Fig. 2d,f). DLAN also increased hippocampal *IL-6* expression during the night (ZT 13, 17 and 21; Bonferroni post-hoc test, *p* < 0.01, Fig. 2e).

### 24-hour variation in interleukin mRNA expression in peripheral tissues

**Figure 3:**
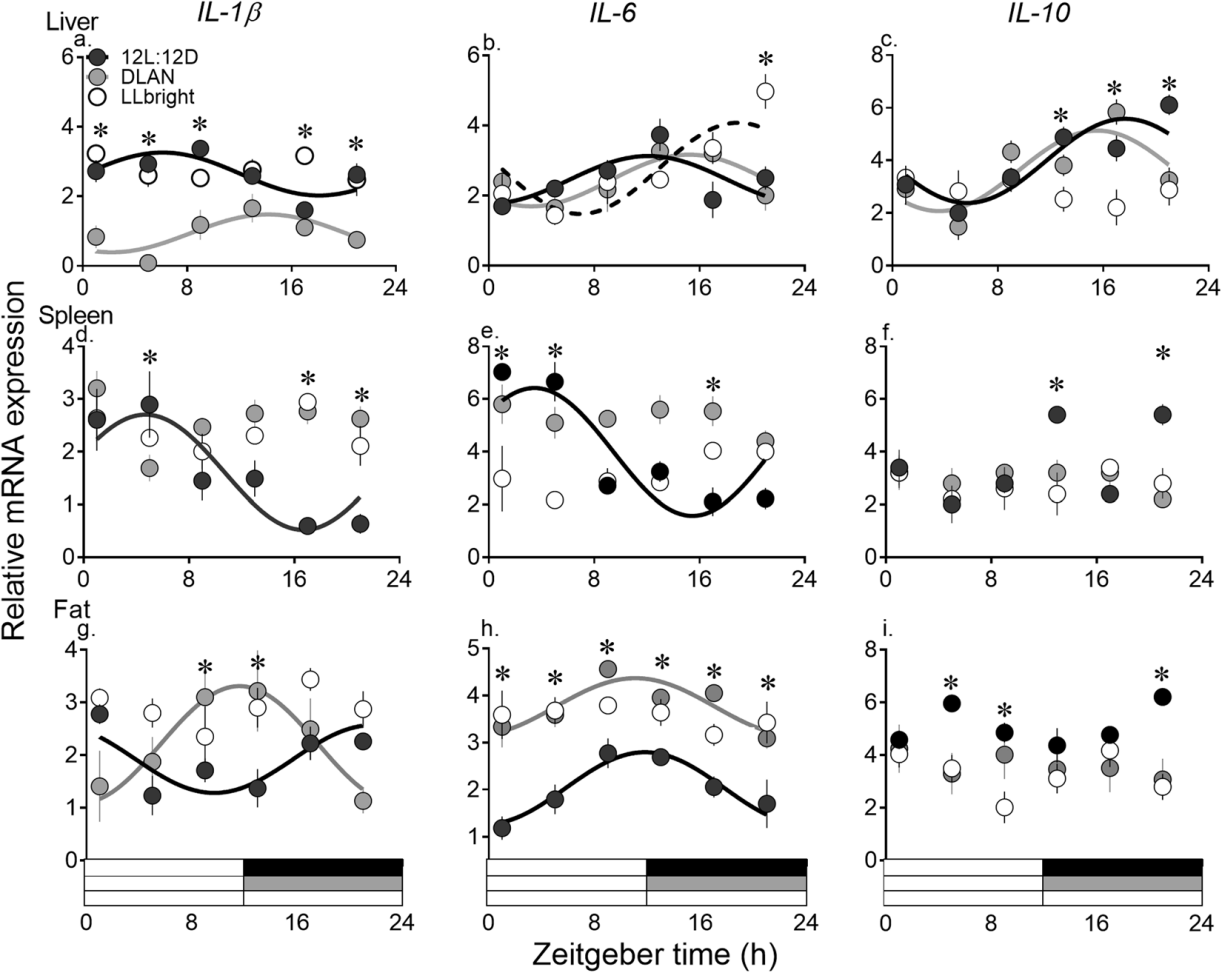
Mean (±SE) mRNA expression levels of genes coding for interleukins (*IL-1β*, *IL-6* and *IL-10*) measured at ZT1, 5, 9, 13, 17 and 21 (Zeitgeber time 0 (ZT 0) = lights on; n = 5/time-point) in liver (**a**–**c**), spleen (**d**–**f**) and fat (**g**–**i**) of zebra finches exposed to 12 L:12D (solid black circles), DLAN (grey solid circles) and constant light (LLbright; white solid circles). Solid black, grey and dotted black curves indicate a significant 24-h rhythm under 12 L:12D, DLAN and LLbright, respectively, as determined by cosinor analysis. (*) Asterisk over time points indicates a significant difference in gene expression under DLAN and/or LL from 12 L:12D, as determined by Bonferroni post-hoc test following a significant LD cycle x time-of-day interaction effect determined by 2-way ANOVA. *p* < 0.05 was considered statistically significant.

All genes assessed in this study were significantly rhythmic under 12 L:12D except *IL-10* in spleen and fat (Cosinor analysis, F-test; Fig. 3, Table 2). Under DLAN, a significant oscillation persisted in all three genes in liver, and in *IL-1β* and *IL-6* in fat (Cosinor analysis, F-test; Fig. 3a–c,g–h, Table 2). Under LLbright, 24-h expression levels of all genes were arrhythmic, except *IL-6* in liver (Cosinor analysis, F-test, Fig. 3b, Table 2). Splenic *IL-1β* and *IL-6* expression under 12 L:12D peaked early during the day (ZT 3–4) (Cosinor analysis, Fig. 3d,e, Tables 2, 3). DLAN exposure shifted *IL-1β* peak expression times to early in the night (ZT 14), as opposed to middle of the day (ZT 6) under 12 L:12D in liver, and to day-to-night transition times (ZT 12), as opposed to night-to-day transition times (ZT 22) under 12 L:12D in fat (Cosinor analysis, Fig. 3a,g, Tables 2, 3). However, *IL-10* in liver and *IL-6* in fat under 12 L:12D and DLAN had similar acrophases (fat: *IL-6* between ZT 10 to 12; liver *IL-10* between ZT 16 to 19; Cosinor analysis, Fig. 3c,h, Table 1). Further, LLbright shifted the peak expression times of *IL-6* in liver to late in the night (ZT 20) as opposed to light/dark transition times in 12 L:12D (ZT 13) and early in the night under DLAN (ZT 15; Cosinor analysis, Fig. 3b, Tables 2, 3).

Overall, effects of LLbright and DLAN on peripheral interleukin mRNA expression were time-of-day dependent, as determined by a significant effect of LD x time-of-day interaction (2-way ANOVA; Table 1; Suppl Table 1). Exposure to LLbright significantly altered hepatic interleukin gene expression with significantly lower expression levels of *IL-10* (at ZT 13, 17 and 21) and significantly higher expression levels of *IL-1β* (at ZT 17) and *IL-6* (at ZT 21), when compared to 12 L:12D (Bonferroni post-hoc test, *p* < 0.05, Fig. 3a–c). DLAN significantly attenuated the hepatic *IL-1β* and *IL-10* expression with significantly lower *IL-1β* levels at all time-points (except ZT 13 and 17) and lower *IL-10* levels at ZT 21, when compared with 12 L:12D (Bonferroni post-hoc test, *p* < 0.05, Fig. 3a–c). Exposure to light pollution induced a pro-inflammatory state in spleen, especially during the night. There was a significant reduction in splenic expression levels of *IL-10* during the night (ZT 13 and 21), and an increase in *IL-6* under DLAN (at ZT 17) and *IL-1β* under DLAN and LLbright (at ZT 17 and 21; Bonferroni post-hoc test, *p* < 0.05, Fig. 3d–f). The day levels of pro-inflammatory interleukin mRNA expression levels were significantly affected, with lower *IL-1β* and *IL-6* at ZT 5 under LLbright and DLAN, and lower *IL-6* at ZT 1 under LLbright, when compared to 12 L:12D (Bonferroni post-hoc test, *p* < 0.05, Fig. 3d,e). In addition, light pollution induced a pro-inflammatory state in adipose tissue, with a significant increase in *IL-6* all throughout the day under DLAN and LLbright (except ZT 9 and 13) and an increase in *IL-1β* expression at light-dark transition times (ZT 9 and 13) under DLAN. There was a significant reduction in *IL-10* expression under DLAN and LLbright at ZT 5, 13 (only LLbright) and 21 (Bonferroni post-hoc test, *p* < 0.05, Fig. 3g–i).

## Discussion

There is accumulating evidence that prolonged exposure to light-at-night (LAN) leads to negative health and fitness consequences^6–11^. In this study, we tested the hypothesis that exposure to LAN alters the daily patterns of cytokine gene expression in addition to key hormones implicated in the regulation of immunity. Our results demonstrated that LAN influences the diel patterns of cytokine gene expression and blood levels of melatonin and corticosterone, along with locomotor activity (cf. Suppl. Fig. 1) in a diurnal avian species. Taken together, our results suggest a more severe effect of bright LAN than dim LAN on tissue-specific daily rhythms of immunity in zebra finches.

The night hormone melatonin accurately encoded the duration of dark (and hence light) with higher night levels than day in zebra finches under 12 L:12D. A peak of melatonin rhythm at ZT 19 is consistent with a mid-night peak in captive zebra finches^3,39^. Furthermore, a significant reduction in night-time melatonin levels to near-day time levels along with an arrhythmic profile under LLbright was expected given the light sensitivity of the melatonin biosynthesis pathway^27^. Thus, an absence of a daily rhythm in melatonin under LLbright was reflective of a lack of physiological differentiation between day and night and hence a state of circadian arrhythmicity^39,40^. Interestingly, the DLAN-induced suppression of melatonin was not as profound as under LLbright. A reduction of melatonin levels at light/dark transition times has also been reported in European blackbirds (*Turdus merula*) exposed to DLAN^41^. We speculate that similar mid-night melatonin levels under pitch-dark and DLAN, and reduced early- and late-night levels under LLbright and DLAN, perhaps indicate that the synchronization of daily rhythms depends on the absolute light intensity as well as the relative interpretation of the phases of illumination based on the photophase contrast^9,40^.

On another level, LAN affects the circadian system through modulation of the hypothalamic-pituitary-adrenal (HPA) axis. In this study, a peak in baseline plasma cort levels towards the end of the 24 h day under 12 L:12D was similar to those reported in other avian species, including Gambel’s white-crowned sparrows^33^, starlings (*Starnus vulgaris*^42^) and tropical Nazca boobies (*Sula granti*^43^). This end-of-night cort peak possibly anticipates higher energy demands and alertness associated with the upcoming active phase for diurnal finches^44,45^. However, end-of-night cort levels in DLAN were significantly lower than those under pitch dark, which was contrary to our expectation of finding elevated cort levels, perhaps owing to circadian disruption, sleep disturbances or restlessness under LAN^7,9,35^. This absence of a night-time elevation in cort under DLAN is consistent with those reported in mammals^46^, but not in free-living birds^35^. This discrepancy could be attributed to a number of factors that include variation in light intensity, species differences, and/or comparing birds from captive and field studies. However, under aperiodic cycles, zebra finches, much like great tits, showed an elevated baseline cort response^35^, along with a non-normal, arrhythmic diel pattern as reported in mice^47^. Thus, 10 days of exposure to ALAN, both bright and dim in intensity, could significantly disrupt diel patterns and alter baseline levels of two important hormones that regulate immunity.

Similar to the endocrine axis, key parameters of the immune system, including cytokines have been shown to exhibit circadian rhythms in mammals^48,49^ and to a lesser degree in birds^21^. Zebra finches entrained to 12 L:12D exhibited significant daily rhythms in transcripts encoding *IL-1β* and *IL-6*, the pro-inflammatory cytokines which function as key regulators of the early immune response to infection^50^ and for *IL-10*, an anti-inflammatory cytokine which dampens inflammatory responses^51^ (cf. Figures 2 and 3). Peak expression of pro-inflammatory cytokines during the light phase or in alignment with the dark-to-light transition (eg. *IL-1β* in fat) in peripheral tissues, and a parallel reduction in anti-inflammatory cytokines (except in fat) is conceivable due to the increased demand of immunity during light hours when birds have a higher risk of exposure to infections when feeding, exploring, and interacting with other individuals^21,52^. However, microglia, the primary innate immune cells of the brain, exhibit diurnal rhythms in inflammatory potential with a peak response generally occurring during the inactive phase of the organism^38,53^. Similar to previous reports, pro- and anti-inflammatory cytokine gene expression in the brain mostly peaked during the dark phase under 12 L:12D with an early night peak of *IL-1β* in hippocampus, a late night peak of *IL-6* and *IL-10* in nidopallium and hypothalamus, and an end-of-the-night peak of *IL-6* in hippocampus (cf. Table 1). Although not established in the current study, this may suggest a sleep regulatory function of neural cytokines^54^ in zebra finches. Further, unlike in rats, where *IL-1β* mRNA peaked just after lights–on in cortex, hippocampus and hypothalamus^55^, we did not find a significant rhythm in *IL-1β* expression in nidopallium and hypothalamus, similar to the absence of day-night variation recently reported in zebra finches^19^. However, we would like to caution that there is a possibility of expression levels in a small nucleus was masked by other nuclei in the brain region, as the mRNA levels were tested in whole hypothalamus, hippocampus and nidopallium.

Given the increasing evidence that neuroendocrine systems regulate immunity and vice versa^56^, we expected an abolishment of diel rhythms in hormone profiles would also induce aperiodic gene expression of cytokines. This was indeed the case except for significant oscillations of *IL-6* in hypothalamus and liver under LLbright. In fact, diurnal variations in microglial inflammatory responses are shown to be entrained by rhythmic expression of glucocorticoids in mammals^53^. Hence, a concurrent aperiodicity in immune response and its endocrine mediators in the present study were expected. However, the physiological significance of a rhythmic hypothalamic and hepatic *IL-6* expression under LLbright remains to be ascertained in future studies. While peripheral metabolic rhythms are known to be entrained independent of the master circadian clock^57^, it is unlikely that metabolic entrainment caused significant hepatic immune rhythm persistence under LLbright since food was available *ad libitum* and food replenishment times were randomized across the 24 hour day (cf. methods section). In comparison to pro-inflammatory cytokine gene expression under LD, elevated night levels of *IL-6* in hypothalamus and liver under LLbright, and perhaps the elevation of *IL-6* in hippocampus as well as *IL-1β* and *IL-6* in fat under DLAN were indicative of a night light-induced immune response^58^. Sleep loss has been shown to increase microglial activity and pro-inflammatory cytokine release in the hippocampus of rats^59^. Such sleep loss induced elevation in neuroinflammation is associated with neurological damage and cognitive decline^60^. However, it is interesting to note that elevated proinflammatory response (*IL-6*) to LLbright and DLAN was evidenced in hypothalamus and hippocampus, but not in cortex, suggesting effects of environmental perturbations on neuroinflammation varied across brain regions. Further studies are warranted to understand how LAN affects neuroinflammation across different brain regions. Also, an upregulation of proinflammatory cytokines in fat and liver in response to LLbright may be attributed to the infiltration of classical proinflammatory M1 macrophages into adipose tissues^22^ and an increased activity of Kupffer cells, the resident tissue macrophages of the liver^25^. Mammalian studies suggest an association of a heightened state of inflammation in adipose tissue with an increased macrophage infiltration in fat of obese individuals. Macrophage infiltration has been shown to alter insulin sensitivity locally in the adipose tissues and systemically^61^. If a proinflammatory state of adipose tissue alters its metabolic and endocrine sensitivity in birds, similar to those reported in mammals, then the light pollution may severely affect fitness in birds, especially species heavily relying on lipid catabolism as a primary energy source during long migratory flights^62^. Further, a chronic pro-inflammatory hepatic response is associated with a variety of physiological and pathophysiological conditions, including hepatic infections, fatty liver disease, liver injury, and fibrosis in mammals^25^. The spleen is involved in humoral and cellular immune responses through its role in generation, maturation and storage of lymphocytes. Cytokine gene expression in the avian spleen is commonly used as an indicator of immune response^20^. Consistent with a previous study reporting increased splenic inflammation under aperiodic conditions^63^, the night time expression of pro-inflammatory cytokines in spleen were elevated under LLbright and DLAN. We would also not rule out a circadian disruption in splenic response to a stimulus (e.g., lipopolysaccharide injection) as reported in other studies^21^.

Studies in diurnal mammals^64^ and domesticated birds^58^ suggest that perturbations in LD cycle tend to alter pro-inflammatory responses in brain and peripheral tissues, perhaps through modulation of melatonin release^15^. Immununoenhancing properties of melatonin are well documented^15^. Melatonin administration rescues the immunosuppressive effects of cortisol in humans^65^. Thus, significant downregulation of cytokine expression in nidopallium and hippocampus under LLbright may be attributed to dampened melatonin and elevated cort responses. However, a lack of similar response in other tissues, especially peripheral tissues, exemplifies the complexities of neuroendocrine-immune (NEI) interactions^56^. It is probable that inflammatory responses from an aperiodic light environment are not solely mediated by melatonin and/or cort but rather by their interaction with other hormones. For example, stimulatory effects of melatonin on cellular and humoral immune response in quail are dependent on opioids^18^, while modulation of seasonal immunity in Indian tropical birds, *Perdicula asiatica*, involves an interaction between melatonin and sex steroids^32^.

Furthermore, since the magnitude and duration of the nocturnal increase in melatonin acts as an entrainment cue for biological functions^27,40^, we hypothesized that the effects of LAN intensity on melatonin would also be reflected in cytokine gene expressions of zebra finches. Indeed, in comparison with LLbright, DLAN-induced effects on diurnal rhythms of cytokines were less apparent. Pro-inflammatory cytokines in liver and fat, *IL-6* in nidopallium and hippocampus, and *IL-10* in liver and hippocampus were significantly rhythmic under DLAN, although there were significant changes in waveform parameters. DLAN is known to alter metabolic state through a shift in timing of food intake^66^. Perhaps, persistence of diurnal variation of the three interleukins in liver, a key metabolic organ, with shifts in waveforms may be attributed to a change in daily activity and feeding patterns. Zebra finches exposed to DLAN were more active during the dark phase than birds in pitch dark in the present study (Suppl Fig. 1). However, an association of behavioral rhythms with immune system need to be ascertained in future studies. Such DLAN induced changes in phase relationships between immune rhythms and other physiological and behavioral rhythms could be speculated to have adverse fitness consequences in the wild, but further study is warranted.

To our knowledge, this is the first study examining tissue-specific alterations in daily oscillations in immune responses in an avian species exposed to LAN. While further study is required to identify a cause-and-effect relationship between endocrine and immune axes, we document concurrent alterations in rhythms of cytokines and their key endocrine regulators that are dependent upon night-light intensity. The alterations of endocrine and immune daily rhythms in the present study were in response to a relatively brighter LAN (3 lux) intensity. We speculate that the diurnal rhythms would show LAN-intensity and -wavelength dependent alterations. A dose dependent advance in activity and suppression of melatonin daily rhythm to LAN has been shown in great tits, *Parus major*^9^ and histological and molecular analyses on testes of great tits indicated a dose-dependent reproductive response to ALAN, with the higher effect on spermatogenesis under 5 lux than under 0.5 and 1.5 lux^67^. Further, zebra finches exposed to 5000 K LAN had increased corticosterone levels compared to finches exposed to 3000 K^36^. It should be noted that we did not test for the ‘free’ endogenous circadian rhythms of cytokines under constant dim light (LL_dim_). However DLAN-induced waveform alterations and LL-induced rhythm disruption suggest diel changes in inflammatory cytokine production may contribute to circadian control of immunity in this diurnal avian species. Future studies are needed to determine whether cytokine rhythm perturbations from LAN are mediated through direct effects of light by a central circadian clock or via its effects on local clock machinery of immune cells. In addition, the results of this study provide an impetus for researchers to evaluate other components of immune function (cellular and humoral responses) that may be differently affected by light perturbations. Lastly, elucidating the adaptive function of these rhythms will increase our understanding of effects upon host fitness and response to disease in a world that is increasingly being affected by light pollution.

## Materials and Methods

### Animals and maintenance

Adult zebra finches (n = 90; 36 females and 54 males) were housed in an indoor flight aviary (96” x 72” x 72”), 12:12 h light-dark cycle, lights on at 8am, 21 °C ± 1 °C) at Western Kentucky University, Bowling Green, Kentucky. We used male and female birds that were acclimated to 12 L:12D cycles for 10 days after purchase from an aviculturist in Washington state. This study was conducted under the approval of the Institutional Animal Care and Use Committee at Western Kentucky University, and procedures followed the National Institutes of Health’s “Guide for the Use and Care of Laboratory Animals” and international ethical standards. Birds were provided with seed, lettuce, grit, cuttlebone and water *ad libitum* throughout the acclimation and experiments.

### Experiment

After acclimating to 12 L:12D, birds were transferred to cages (n = 5/cage; 34 cm × 40 cm × 45 cm) and exposed to light:dark cycles: (a) 12 h light:12 h darkness (12 L:12D), (b) 12 h light:12 h dim light at night (12 L:12L_dim_, DLAN) or (c) constant light (24 L:0D, LLbright). Each cage i.e. each sampling time point consisted of 3 male and 2 female birds. Thus, 6 cages (30 birds; 18 male and 12 females) were sampled per experimental treatment with each cage representing one time point. Experiments were sequentially performed: after termination of the 12 L:12D experiment, the LLbright experiment was conducted, which was followed by the DLAN experiment. We recognize that housing individual birds in single cages would obviate a potential issue with cage environment (i.e., social dynamics). However, given space limitations within our animal facilities, this was not feasible. Therefore, we cannot technically rule out that the some of the variation reported herein was influenced by cage effects. However, the experiments were performed in the same experimental room and in identical-sized cages and housing conditions to ensure minimal variability across experiments. White fluorescent bulbs (Philips 281030) with an intensity of 400 ± 50 (mean ± SD) lux was the source of light during the 12 L of 12 L:12D and DLAN, and during 24 h of LLbright. The night of 12 L:12D and DLAN was pitch dark and blue light of 3 ± 1 (mean ± SD) lux, respectively, as measured with luxmeter (Digi-sense data logging light meter, model 20250-00) at the level of both perches (upper and lower) in each cage.

We determined the effects of light:dark cycles on the 24-h oscillation of interleukin genes (*IL-1β*, *IL-6* and *IL-10*) in three brain regions (nidopallium; hippocampus; hypothalamus) and peripheral (liver, spleen and fat) tissues of zebra finches. Birds were euthanized at 4 h intervals, beginning 1 h after light on (Zeitgeber time, ZT 0 = time of lights on) at the end of 10 days of exposure to one of the three light:dark cycles. Thus, birds were sampled at ZT 1, 5, 9, 13, 17 and 21 on day 11 of 12 L:12D, DLAN or LLbright exposure, with n = 5 (1 cage) contributing to each sampling point. At each sampling time point, birds were first bled from the wing vein within 2 min of capture and then decapitated under the brief influence of isoflurane vapors (<10 s). Blood from the wing vein and trunk blood from decapitation were collected, kept on ice for <20 min, and then spun at 3000 g for 30 min at 4 °C. The plasma from wing vein blood and serum from decapitation were drawn out and stored at −20 °C for later ELISA of corticosterone (cort) and melatonin, respectively. For the gene expression studies, liver, spleen, fat, and brain were dissected out from decapitated finches and stored in RNAlater solution (ThermoFisher Scientific; Catalogue number: AM7020) at 4 °C until used for RNA extraction. For dissecting out nidopallium, hippocampus and hypothalamus, the brain was excised from the skull and placed dorsal side down in a petri dish. The diencephalon was separated out by making two coronal incisions on either side of the optic-chiasma. From this brain slice, the hypothalamus was removed roughly in the shape of an inverted V (Λ) by longitudinal incision placed at 45° angle on either side of the third ventricle^68^. From the same slice of brain, the cerebral part was sectioned to dissect out the hippocampus and nidopallium. Both left and right hippocampal formations were collected. While care was taken to not include any other part of cortex with the hippocampal tissue, fractions of para hippocampal area (APH) were included in the hippocampus samples due to the irregularity in locating the paraventricular sulcus^69^. As an analogue of mammalian pre-frontal cortex (PFC), nidopallium, located ventral to APH and adjacent to the fourth ventricle, was excised out consistently from the right cerebral hemisphere from all birds^70^. A representative picture of dissection is provided (Supplementary Fig. 1).

### Measurement of gene expression

RNA was extracted from nidopallium, hippocampus, hypothalamus, liver, spleen and fat^19,68^ using a RNeasy mini kit (Qiagen). A NanoDrop 2000 Spectrophotometer (ThermoScientific) was used to measure total RNA concentrations and a high-capacity cDNA reverse transcription kit (Life Technologies, Cat number: 4368813) was used to reverse transcribe total RNA into cDNA. The prepared cDNA was used as a template for determining relative cytokine gene expression using an ABI 7300 RT-PCR machine. Cytokine probes (Table 4; Applied Biosystems) labelled with fluorescent marker 5-FAM at the 5′ end and quencher MGB at the 3′end were used, along with PPIA VIC-labelled probe as endogenous control (house-keeping gene^71^) according to the manufacturer’s instructions. Samples were run in duplicate, and the values from duplicates were averaged for each gene. ΔCt values were calculated from cycle threshold (Ct) values of target and endogenous control gene expression as follows: (Ct[target gene] - Ct[PPIA]). Each ΔCt value was normalized against the lowest ΔCt value of ZT1^68,72^ for calculation of ΔΔCt. 2^−ΔΔCt^ to measure relative mRNA expression levels.

### Corticosterone and melatonin ELISA

Corticosterone immunoassay kits from Enzo Life Sciences (Ann Arbor, MI; cat. no. ADI-900-097) were used to measure plasma cort levels in zebra finch plasma in a 96-well plate as per the manufacturers’ protocol^73^. All samples (in 1:40 dilution in assay buffer and 1% steroid displacement buffer) and standards were run in duplicate. Samples were spread across three ELISA plates to ensure equal distribution of time points across assays (two assays comprised of 2 samples from each time point/LD condition and a third assay comprised of 1 sample/time point/LD condition). Inter- and intra-assay variations were 8.19% and 13.37%, respectively. The plates were read at 405 nm on a Synergy H1 hybrid microplate reader (BioTex Instruments, Vermont, USA).

Plasma melatonin levels were measured in the serum extracted from trunk blood samples using an immunoassay kit from Aviva Systems Biology (San Diego, California; cat. no. OKEH02566) as per the manufacturers’ protocol. Using a pool of zebra finch plasma for a test of parallelism, our lab was able to validate the use of this kit to measure melatonin in this species (Suppl. Fig. 1). Briefly, we ran the assay using 50 μl serum samples per well. Samples and standards were run in duplicate. The plate was read at 450 nm on a Synergy H1 hybrid microplate reader (BioTex Instruments, Vermont, USA). Inter- and intraassay variation of the ELISA run were 13.72% and 10.94%, respectively.

### Locomotor activity

To ensure that 10 days of exposure to light:dark cycles sufficiently altered behavioral rhythms, 24-h locomotor activity was recorded on day 11 of 12 L:12D, DLAN, or LLbright exposure (n = 2/group). Activity profiles were assessed using video tracking software (Limelight, Coulbourn Instruments, Holliston, MA) to assess movement of finches in individual cages. Movement was tracked 7.5 frames per second, and the distance travelled (cm) was recorded and binned into 10-min intervals for 24 h (Suppl. Fig. 1).

### Statistics

Data are presented as mean (±SE). The effects of light-dark cycle (LD; factor 1), time of day (factor 2) and their interaction (factor 1 x factor 2) were assessed using two-way analysis of variance (2-way ANOVA). Bonferroni post-hoc tests were used for comparison of LD cycles at each time point if 2-way ANOVAs revealed significant differences. The strength of a significant effect was further validated by effect size estimate (partial eta^2^) and observed power in a 2-way ANOVA (Supplementary Table 1). Unimodal cosinor regression analysis (y = A + [B.cos (2π (x-C)/24)]) was used to assess the daily rhythms of 24-h mRNA expression levels and hormone levels. Mean (mesor), amplitude, and acrophase of the cosinor regression is denoted as A, B and C, respectively, and ‘cos’ represents the cosine trigonometric function^68,72^. Numbers of predictors, i.e. the amplitude, acrophase and mesor, R^2^ values, and number of samples were used (http://www.danielsoper.-com/statcalc3/calc.aspx?id=15)^74^ for calculating the significance of cosinor regression analysis. Goodness of Cosinor fit was also validated with the degree of freedom (Df), absolute sum of squares (SS), standard deviation of the residuals (Sy.x), Akaike information criterion (AICc) values (Supplementary Table 2), in addition to the R square values (R^2^, Table 2). The three rhythm waveform parameters (mesor, amplitude and acrophase) were used to show differences in daily rhythm between light conditions and/or tissues. Mesor defined the baseline mRNA expression levels, amplitude defined the maximum change in mRNA expression, and acrophase defined the estimated time of peak mRNA expression levels. The zeitgeber hours of 12 L:12D served as a time reference to depict 24-h mRNA and hormone profiles, since we did not have defined onsets or offsets during LLbright. The statistical analysis was performed using IBM SPSS statistics software (version 20; IBM Corp., Armonk, NY, USA) and GraphPad Prism software (version 6.0; GraphPad Software, La Jolla, CA, USA). Alpha for statistical significance was set at 0.05. We transformed gene expression values by dividing all 2^−ΔΔCt^ values by the lowest 2^−ΔΔCt^ value. Hence, the lowest 2^−ΔΔCt^ value was 1 and its log2 value = 0, and the change in y- axis scale from 0 to 1 indicates a 2-fold rise in mRNA expression and from 0 to 2 indicates a 4-fold rise^75^. This was done for better visual demonstration of gene expression change.

## Supplementary information

Supplementary Info

## Data Availability

The datasets generated during and/or analyzed during the current study are available from the corresponding author on reasonable request.
