# Supplementary material for: Light at night disrupts diel patterns of cytokine gene expression and endocrine profiles in zebra finch (Taeniopygia guttata)
Source: Sci Rep. 2019 Nov 1;9:15833. doi: 10.1038/s41598-019-51791-9 (PMC6825233; doi:10.1038/s41598-019-51791-9)
Supplement: Supplementary file 1 — Supplementary Info [file 41598_2019_51791_MOESM1_ESM.pdf]

Supplementary Information

Light at night disrupts diel patterns of cytokine gene expression and endocrine profiles in zebra finch (*Taeniopygia guttata)*

Ila Mishra[1], Reinhard M. Knerr[1], Alexander A. Stewart[1], Wesley I. Payette[1], Melanie M. Richter[1], Noah T. Ashley[1]

1: Department of Biology, Western Kentucky University, Bowling Green, KY, USA

* Corresponding author

Email: noah.ashley@wku.edu

Supplementary Figure 1: (a) Standard curve validation of melatonin ELISA kit. Elisa kit standards and night-time plasma samples of zebra finch were serially diluted 1:1 to test for parallelism. (b) Shows isolation of brain tissue for qPCR. Solid lines depict the plane of coronal cuts made to visualize the hypothalamic region. Dotted lines on right shows the hypothalamic region around the third ventricle and above the optic-chiasm that was excised out. From the same slice of brain, the cerebral part was sectioned to dissect out the hippocampus and nidopallium. Both left and right hippocampal formations were collected. Nidopallium, located ventral to APH and adjacent to fourth ventricle, was excised out consistently from the right cerebral hemisphere from all birds. The dissections were performed with brain put in a petri dish cleaned with RNAZap and kept on ice. All surgical instruments were cleaned with RNAZap in between brain dissections. (c) Heat map showing 24-h activity profiles (n = 2/group) assessed using video tracking software (Limelight, Coulbourn Instruments, Holliston, MA) to assess movement of finches in individual cages. Note an increased night-time activity of birds exposed to DLAN and arrhythmic activity pattern of birds under LLbright.

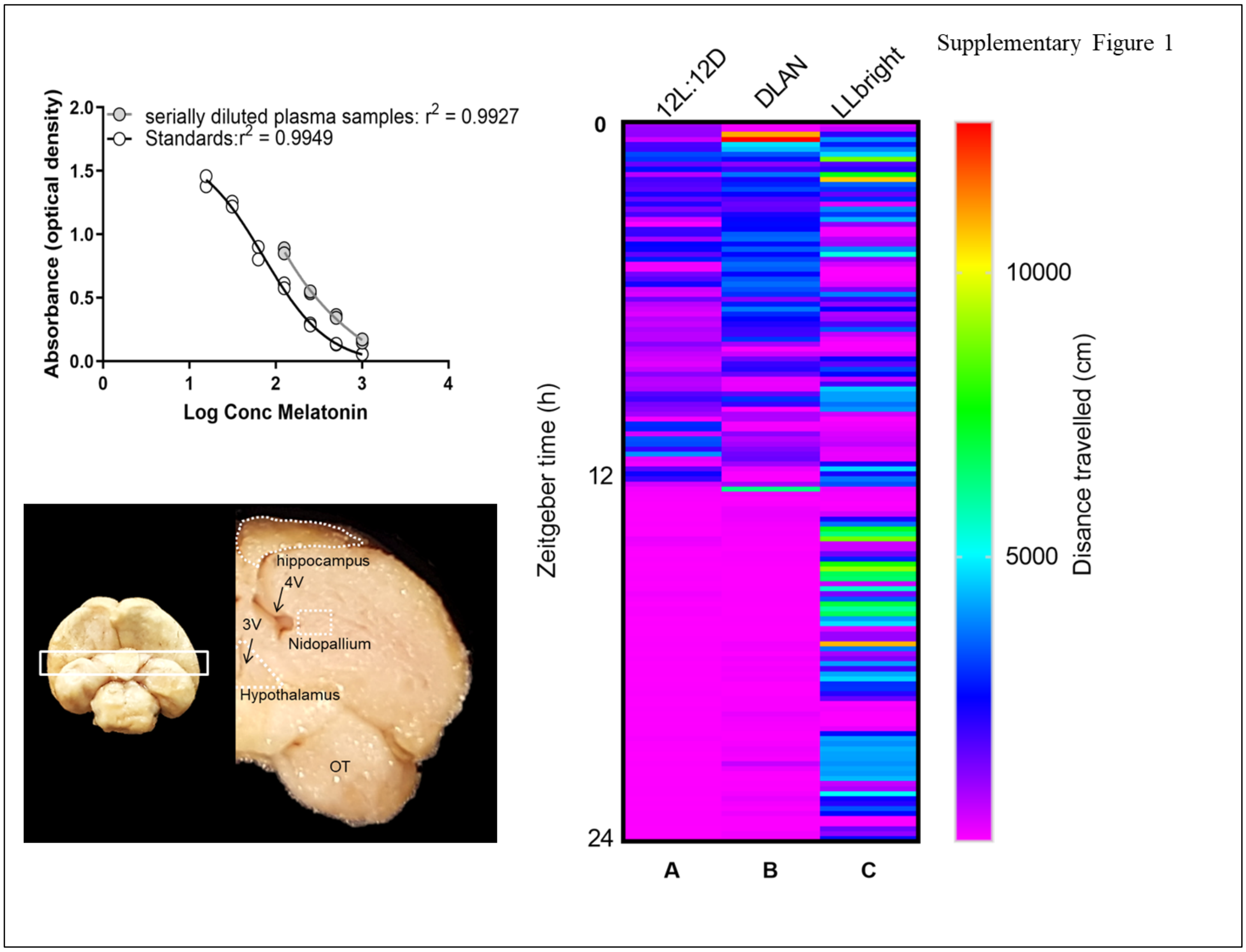

Table S1: Effect size estimate (partial eta^2) and observed power of Two-way Analysis of variance (2-way ANOVA) testing the effect of light:Dark (LD) cycle (factor 1), Time-of-day (factor 2) and their interaction (factor 1 x factor 2) on plasma hormone levels, and interleukin gene expression profile in tissues (Suppl. Information to Table 2).

Tissue	Parameter tested	Light: Dark cycle (Factor 1)		Time-of Day (Factor 2)		Factor 1 x Factor 2	
		Partial eta^2	Observed Power	Partial eta^2	Observed Power	Partial eta^2	Observed Power
Blood	Melatonin	0.14	0.83	0.25	0.96	0.20	0.75
	Cort	0.09	0.63	0.17	0.82	0.31	0.98
NCL	*IL-1β*	0.30	0.99	0.14	0.69	0.33	0.99
	IL-6	0.46	1.00	0.04	0.22	0.42	1.00
	IL-10	0.53	1.00	0.32	0.98	0.46	0.99
Hippocampus	*IL-1β*	0.64	1.00	0.15	0.74	0.25	0.91
	IL-6	0.41	1.00	0.18	0.85	0.28	0.95
	IL-10	0.58	1.00	0.33	0.99	0.46	1.00
Hypothalamus	*IL-1β*	0.33	1.00	0.18	0.85	0.26	0.92
	IL-6	0.50	1.00	0.22	0.92	0.38	0.99
	IL-10	0.62	1.00	0.60	1.00	0.77	1.00
Liver	*IL-1β*	0.52	1.00	0.07	0.35	0.31	0.97
	IL-6	0.16	0.91	0.36	0.99	0.55	1.00
	IL-10	0.19	0.95	0.31	0.99	0.51	1.00
Spleen	*IL-1β*	0.09	0.63	0.20	0.91	0.30	0.97
	IL-6	0.25	0.99	0.40	1.00	0.41	0.99
	IL-10	0.27	1.00	0.30	0.99	0.52	1.00
Fat	*IL-1β*	0.22	0.98	0.15	0.74	0.38	0.99
	IL-6	0.54	1.00	0.20	0.90	0.23	0.86
	IL-10	0.48	1.00	0.13	0.68	0.50	1.00

Table S2: Significance of diurnal rhythm in plasma melatonin and tissue-specific gene expression tested using cosinor regression. Goodness of fit of cosinor regression curve presented as degree of freedom (Df), absolute sum of squares (SS), standard deviation of the residuals (Sy.x), akaike information criterion (AICc) values. R square values (R^2) are reported in Table 3.

Tissue	Parameter tested	Light: Dark cycle	Goodness of Fit			
			Df	SS	Sy.x	AICc
Blood	Melatonin	12L:12D	27	2177.24	91.5	268.5
	Cort	12L:12D	27	285.9	3.25	77.23
NCL	*IL6*	12L:12D	27	4.11	0.39	-50
		DLAN	27	5.11	0.44	-43.52
	IL-10	12L:12D	27	1.36	0.24	-66.7
Hippocampus	*IL-1β*	12L:12D	27	1.46	0.23	-81.1
	IL-6	12L:12D	27	0.86	0.18	-97
		DLAN	27	4.68	0.42	-43.25
	IL-10	DLAN	27	1.44	0.23	-81.55
Hypothalamus	*IL-6*	12L:12D	27	1.75	0.26	-71.84
		LLbright	27	4.98	0.44	-41.41
	IL-10	12L:12D	27	429.4	3.99	89.44
Liver	*IL-1β*	12L:12D	27	1.11	0.20	-89.16
		DLAN	27	0.19	0.08	-142.5
	IL-6	12L:12D	27	3.97	0.38	-51.05
		DLAN	27	1.87	0.27	-69.89
		LLbright	27	27.40	1.00	6.89
	IL-10	12L:12D	27	32.72	1.10	12.21
		DLAN	27	30.49	1.06	10.08
Spleen	*IL-1β*	12L:12D	27	1.42	0.23	-81.95
	IL-6	12L:12D	27	2.02	0.27	-71.62
Fat	*IL-1β*	12L:12D	27	0.72	0.17	-92.73
		DLAN	27	6.96	0.51	-34.22
	IL-6	12L:12D	27	0.18	0.08	-143.7
		DLAN	27	1.47	0.23	-80.95
